# Development of a colloidal gold immunochromatographic strip with enhanced signal for the detection of bovine parvovirus

**DOI:** 10.3389/fmicb.2023.1174737

**Published:** 2023-05-09

**Authors:** Xiaoli Yu, Yanping Jiang, Songsong Zhang, Caihong Wang, Ruichong Wang, Lanlan Zhang, Siming Tao, Wen Cui, Jiaxuan Li, Xinyuan Qiao

**Affiliations:** ^1^Heilongjiang Key Laboratory for Animal Disease Control and Pharmaceutical Development, Department of Preventive Veterinary Medicine, College of Veterinary Medicine, Northeast Agricultural University, Harbin, China; ^2^Department for Radiological Protection, Heilongjiang Province Center for Disease Control and Prevention, Harbin, China; ^3^Promotion Demonstration Department of Heilongjiang Fishery Technology Extension Station, Harbin, China

**Keywords:** bovine parvovirus, monoclonal antibody, signal enhancement, colloidal gold immunochromatography, antigen dectection

## Abstract

Bovine parvovirus (BPV) is a pathogen responsible for respiratory and digestive tract symptoms in calves and abortion and stillbirth in pregnant cows. In this study, we developed a colloidal gold immunochromatographic (GICG) strip with an enhanced signal for detecting BPV according to the double-antibody sandwich principle and an enzyme-based signal amplification system to amplify the signal. This system utilizes horseradish peroxidase reacting with a substrate solution containing 3,3′,5,5*′*-tetramethylbenzidine and dextran sulfate to obtain insoluble blue products on the test and control lines. We optimized different reaction conditions, including the amount of monoclonal antibodies (mAbs), pH of the colloidal gold solution, coating solution, blocking solution, sample pad treatment solution, antibody concentration in the control line, and antibody concentration in the detection line. The sensitivity of the signal-enhanced GICG strip showed that the minimum amount for detecting BPV was 10^2^ TCID_50_, 10 times higher than that of the traditional GICG strip. The results of the specificity test showed that the signal-enhanced GICG strip had no cross-reactivity with BRV, BVDV, or BRSV. The results of the repeatability test showed that the coefficient of variation between and within batches was less than 5%, showing good repeatability. Moreover, for validation, PCR and the signal-enhanced GICG strip were used to detect 280 clinical bovine fecal samples. The concordance rate compared with PCR was 99.29%. Hence, the developed strip exhibited high sensitivity and specificity for the detection of BPV. Therefore, this strip could be a rapid, convenient, and effective method for the diagnosis of BPV infection in the field.

## 1. Introduction

Bovine parvovirus (BPV) is a member of the genus Bocaparvovirus of the family Parvoviridae (Qiu et al., 2017). It is the smallest icosahedral virus, with no envelope and a diameter of approximately 23 nm. The BPV genome consists of single-stranded DNA with a length of 5,491 nt (Kailasan et al., 2015), with three open reading frames (ORFs). ORF1 encodes a 729-amino-acid-long protein, the non-structural protein NS1. ORF2 encodes a 255-amino-acid-long protein, the non-structural protein NP1. The viral structural proteins VP1 and VP2 are encoded by ORF3. The molecular weights of VP1 and VP2 are 75 and 61 kDa, respectively. At the same time, VP3 is produced after the hydrolysis of the VP2 protein, which is involved in BPV DNA replication and virion assembly. BPV infection mainly causes reproductive dysfunction in pregnant cows and respiratory and gastrointestinal diseases in newborn calves. BPV can be transmitted in several ways, and the initial clinical symptoms of infection are not obvious (Zhang et al., 2020), making it difficult to diagnose and prevent infections. Traditionally, BPV detection is mainly based on serological, etiological, and molecular methods. Most traditional methods require a long time and special laboratory diagnosis equipment (Wang et al., 2019a; Gong et al., 2020). Therefore, a rapid, specific, and convenient method for field detection is of practical significance for the prevention and control of BPV infections.

Since colloidal gold has been introduced into the field of immunochemistry, this technology has developed and matured. Specifically, the colloidal gold immunochromatographic (GICG) strip is a fast and convenient detection method that is especially suitable for on-site detection. The reaction of gold-labeled antibodies with their corresponding antigens can result in a visible color reaction (Vilela et al., 2012). The distinctive advantages of colloidal gold particles are that they can be directly observed, and visible results can be obtained. Therefore, GICG strips have been widely used in various applications, Iing the diagnosis of viral infections and the detection of bacteria and drug residues in food (Wu et al., 2017; Lin et al., 2020; Pan et al., 2020). Their rapid analysis and simplicity of operation provide promising diagnostic methods for various applications. However, their detection sensitivity is low, limiting their application in clinical diagnosis. Hence, improving the sensitivity of GICG is an important research direction (Guo et al., 2019, 2021).

Currently, strategies to improve immunochromatographic technologies mainly focus on developing new solid-phase carriers or the addition of novel labeling molecules. Some studies combined newer technologies with strip detection, such as photoelectric sensing and microchip technologies. One strategy to improve sensitivity is to combine an enzyme signal amplification system with colloidal gold-labeled antibodies. Colloidal Au particles can be used to combine antibodies and enzymes. Moreover, horseradish peroxidase (HRP) can be conjugated to colloidal gold-labeled antibodies and improve the color depth of the strip (Parolo et al., 2013; Cho et al., 2015). Hence, enzyme signal amplification systems can effectively improve the detection sensitivity of GICG strips.

In this study, a signal-enhanced GICG strip for detecting BPV was developed using purified monoclonal antibodies (mAbs) and polyclonal antibodies (pAbs) against BPV. This method is specific, rapid, and sensitive for the detection of BPV, which is suitable for pathogen diagnosis in the field.

## 2. Materials and methods

### 2.1. Ethics statement

All animal experiments and animal maintenance procedures were performed according to the Ethical Committee for Animal Sciences of Heilongjiang Province and international recommendations for animal welfare. This trial was conducted in accordance with the regulations governing laboratory animals and the Charter of the Ethics Committee for Laboratory Animals of Northeast Agricultural University (Protocol code NEAU2018024).

### 2.2. Cells and virus strains

The myeloma (SP2/0) cells line was purchased from the China Center for Type Culture Collection (Wuhan, China). Bovine parvovirus(BPV; ATCC strain VR-767), Bovine rotavirus (BRV; strain NCDV), Bovine viral diarrhea virus (BVDV; strain BA), and Bovine respiratory syncytial virus (BRSV; strain 391–2), were stored in our lab. Bovine turbinate (BT) cell lines.

### 2.3. Animals

Specific pathogen-free BALB/c mice and New Zealand rabbits were purchased from Changsheng Biotechnology Limited (Liaoning, China).

### 2.4. The culture and purification of BPV

Maintain BT cells in culture flasks containing cell maintenance medium in an incubator at 37°C with 5% CO_2_. Trypsinize a confluent flask of BT cells, and then trypsin was discarded. After adding 3% v/v serum cell maintenance solution, and cultured it in an incubator for 6–12 h. After that, BPV was inoculated into BT cells. When the cytopathic effect reached 80%, the virus culture was collected, which was centrifuged at 4°C and 10,000 r/min for 2 min. The supernatant fluid containing virus was collected and used to immunize animals.

### 2.5. Preparation of pAbs and mAbs

New Zealand rabbits were administered with purified BPV (500 μg) after emulsification in complete Freunds’ adjuvant (Sigma, St. Louis, MO, United States) for the first injection and the same dose after emulsification in incomplete Freunds’ adjuvant (Sigma) given as two boosters every 2 weeks. Sera were collected 7 days after the last booster. Immunoglobulins were precipitated using standard ammonium sulfate precipitation. Briefly, an equal amount of saturated ammonium persulfate solution was dropped into the mixed serum, stirring on ice until a precipitate was formed. The mixture was centrifuged at 12,000 × *g* for 30 min. The deposits were dissolved in PBS, dialyzed against PBS, and the protein concentration was determined. The pAbs were analyzed using sodium dodecyl sulfate-polyacrylamide gel electrophoresis (SDS-PAGE) and an indirect immunofluorescence assay (IFA). In the IFA test, monoclonal or polyclonal antibodies were used as primary antibodies, and FITC-labeled goat anti-rabbit IgG (Sigma) or FITC-goat anti-mouse IgG (Sigma) were used as the secondary antibody.

BALB/c mice were administered purified BPV (100 ng) after emulsification in complete Freund’s adjuvant for the first injection and the same dose after emulsification in incomplete Freund’s adjuvant for two boosters every 2 weeks. After BALB/c mice were immunized with BPV, the splenocytes were harvested and fused with SP2/0 myeloma cells. After fusion, hybridoma cells were screened using an indirect enzyme-linked immunosorbent assay (ELISA).

BALB/c mice were pretreated with liquid paraffin and then injected intraperitoneally with hybridoma cells secreting antibodies against BPV. Ascites were collected after 2 weeks. A double-antibody sandwich ELISA was used to detect ascites titers. Hybridoma cells with the highest titers and affinities were selected to prepare ascites and purify mAbs for use in subsequent experiments. Ascites were purified using a HiTrap Protein G HP (GE Healthcare, Milwaukee, United States) according to the manufacturer’s instructions. The activity of the mAbs was analyzed *via* SDS-PAGE and IFA identification. MAbs isotype was detected by using an Monoclonal antibody isotyping kit (Sigma). Bovine Parvovirus protein reacting against mAbs was detected by Western blotting. The BT cell culture was as negative control.

### 2.6. Preparation of colloidal gold

Gold particles with an average diameter of 20 nm were prepared according to the method described by Wang et al. (2014). In brief, a suspension of gold particles was prepared under reflux conditions where 100 mL of a gold chloride solution (0.01%) was heated to boiling. Approximately, 1.0 mL of trisodium citrate solution (1%) was then added rapidly to the gold chloride solution while stirring. The resulting solution was boiled for another 5–10 min until the color of the mixture changed to wine red. The gold particles were detected using transmission electron microscopy (TEM) after cooling. Finally, 0.05% sodium azide (as a preservative) was added to the gold particle solution and then stored at 4°C.

### 2.7. Conjugation of anti-BPV mAbs with colloidal gold

Complexes of mAbs conjugated with colloidal gold were prepared according to a previous method (Shukla et al., 2011). In brief, the pH of the colloidal gold solution was adjusted to 8.0–9.0 with 0.2 M K_2_CO_3_. The following procedure was conducted to estimate the minimal amount of mAbs required to stabilize colloidal gold particles. First, 1 mL of colloidal gold solution was added quickly to 100 μL of serial dilutions of mAbs at increasing concentrations (10–100 μg/mL). After 5 min, 100 μL of 10% NaCl solution was added to the mixture and was left to stand for another 2 h. When the amount of mAbs added exceeded the minimum required to stabilize colloidal gold particles, the color was unchanged or changed from reddish to blue. The optimum concentration of mAbs added was 130% of the lowest concentration needed for labeling. One hundred microliters of mAbs at the optimum concentration were then added to each tube and mixed. After 5 min, 100 μL of a 10% NaCl solution was added to the mixture. After 2 h, the color of the solution was observed. The optimum pH of the colloidal gold solution was the minimum pH at which the solution remained reddish.

To conjugate anti-BPV mAbs with colloidal gold, the optimum concentration of mAbs which determined by the above method was rapidly added to 20 mL of the colloidal gold solution and incubated for 30 min after rapid stirring. The mixture was then stabilized with a 5% BSA solution (the final concentration of BSA was 1%) and stirred for 30 min. After incubation for 1 h, the supernatant was discarded *via* centrifugation at 10,000 × g for 30 min at 4°C. Twenty milliliters of a 2% BSA solution (containing 0.01 M sodium borate) was then used to resuspend the precipitate, which was centrifuged again at 10,000 × *g* for 30 min at 4°C to clean off the unlabeled mAbs. Finally, 4 mL of a TB solution (containing 3% sucrose, 3% BSA, 0.05% sodium azide, and 0.01 M sodium borate) was used to resuspend the precipitate. The solution of colloidal gold-labeled mAbs was stored at 4°C. The conjugation of colloidal gold with mAbs was confirmed using UV–visible (UV/Vis) spectroscopy (Ultropec 2100 pro UV; Amersham Pharmacia, Sydney). The gold-labeled mAb solutions were stored at 4°C until use.

### 2.8. Preparation of colloidal gold and enzyme-labeled antibody conjugates

Horseradish peroxidase was used as the labeling enzyme. HRP-labeled mAbs were prepared by diluting the mAbs to 1 mg/mL following the instructions of an antibody-conjugated HRP kit (Abcam, Shanghai, China). ELISA was used to identify the effect of antibody conjugation with HRP. Briefly, the plates were coated with BPV, incubated overnight at 4°C, and blocked with PBS containing 2% BSA. Then, 100 μL of a 1:100 dilution of the enzyme was added to label the mAbs, which were incubated for 1.5 h at 37°C. After washing, 100 μl diluted HRP-labeled goat anti-mouse secondary antibodies (Sigma-Aldrich, United States) were added to the plates and incubated at 37°C for 1.5 h. The plates were washed by deionized water and 100 μL of 3,3′,5,5′-tetramethylbenzidine (TMB) color development solution (Sigma) was added. After incubating at 37°C in the dark for 10 min, 50 μL of a 10% of sulfuric acid was added, and the absorbance was measured at 490 nm using a microplate reader.

Complexes of enzyme-labeled mAbs conjugated with colloidal gold were prepared according to a previous method (Shukla et al., 2011). The prepared HRP enzyme-labeled mAbs were conjugated with colloidal gold particles under the same conditions as previously described, which was placed in the conjugate pad. The conjugation of colloidal gold with mAbs was examined using UV/Vis spectroscopy.

### 2.9. Optimizing the reaction reagents

**Figure 1:**
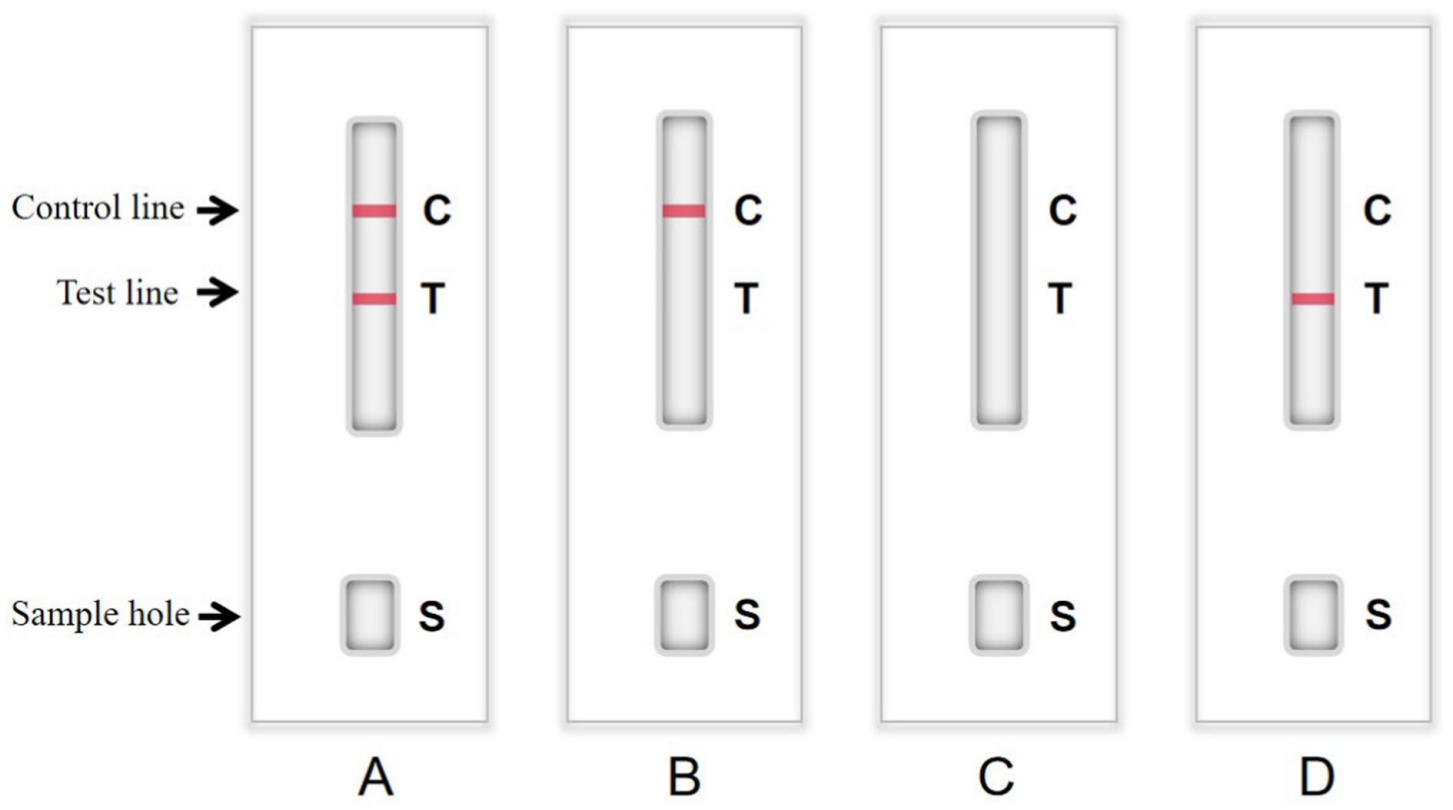
Structure chart of colloidal gold immunochromatographic (GICG) strip. **(A)** The test result is positive when both the test line and the control line are red. **(B)** When only the control line is red, the test result is negative. **(C)** The test result is invalid when there is no strip color. **(D)** The detection result is invalid only when the test line is red.

It is necessary to optimize the reaction reagents to improve the performance of signal-enhanced test strips. Here, 0.01 M phosphate buffer (PB, containing 0.6% NaH_2_PO_4_·2H_2_O and 2.2% Na_2_HPO_4_·12H_2_O), PBS, and 20 mM Tris-Cl were used as coating solutions to treat the antibodies. Control and test lines were used to analyze the effects of these coating solutions. Moreover, 3% BSA, 5% BSA, 3% skim milk, and 5% skim milk were used as blocking solutions, respectively. The background color and sample chromatography time were determined to analyze the effects of blocking. The sample pads were soaked in solution A (0.05% Tween-20, 5% sucrose, 0.3% Triton X-100, and 1% BSA), solution B (0.05% Tween-20, 5% sucrose, 0.5% Triton X-100, and 0.5% BSA), solution C (5% sucrose, 0.3% Triton X-100, and 1% BSA), and solution D (5% sucrose, 0.5% Triton X-100, and 0.5% BSA) at 25°C for 30 min. Afterward, the sample pads were dried at 37°C for 3 h and stored at room temperature. Test lines were used to analyze the effects of the different treatment solutions. The detection procedure was as follows. First, 50 μL of a sample was added to the sample pad. TMB substrate was added to the sample pad, and the NC membranes were rinsed with deionized water. After 10 min, the result was judged using the naked eye. If the sample contained detectable particles, they formed complexes with the enzyme-labeled mAbs conjugated with colloidal gold. If the test line showed a weak color or color, the test result was weakly positive or positive, respectively. If the sample did not contain detectable particles, the test line would be colorless, indicating that the test result was negative. The color of the control line was used as a standard to evaluate whether the prepared strip was valid (color) or invalid (colorless; Figure 1). The goat anti-mouse IgG with a concentration of 1 mg/mL g was coated on the control line. The mAb in the conjugate pad bound with the colloidal gold and HRP conjugate to color the control line during testing.

### 2.10. Sensitivity and specificity of the prepared signal-enhanced test strips

Under the optimized conditions, enzyme-labeled signal-enhanced test strips and conventional test strips without enzyme labeling were prepared simultaneously. The sensitivity of the two test strips was also tested. Samples containing different concentrations of BPV (10^5^ TCID_50_/0.1 mL) were diluted in a series of 1, 1:10, 1:100, 1:1,000, and 1:10,000. The sensitivity of the signal-enhanced test strip was evaluated by detecting BPV at different concentrations, and the results were evaluated using the naked eye. The procedure was repeated thrice.

The specificity test was conducted with standard negative samples, standard positive samples, and samples containing bovine rotavirus (BRV), bovine viral diarrhea virus (BVDV), or bovine respiratory syncytial virus (BRSV) under optimized conditions to evaluate the specificity of the signal-enhanced test strip. Briefly, 50 μL of the samples were added to the sample pad and the results were observed after 10 min using the naked eye. The procedure was repeated thrice.

### 2.11. Detection of clinical samples

A total of 280 fecal samples from different farms where diarrheal disease occurred (Jilin Province, Liaoning Province, and Heilongjiang Province in China) were collected, placed in sterile polyethylene tubes, numbered, and transported to the laboratory using a car freezer (4°C). The fecal sample of 2 g was dissolved in 0.5 mL of sterile PBS solution, which was centrifuged for 10 min at 1,000 r/min, and then the supernatant was dropped in the sample hole of the developed strip. The results were observed after 10 min using the naked eye. The samples were then analyzed *via* PCR, conventional strip test, and the developed strips. We use a DNA extraction kit (Sigma-Aldrich, United States) to extract DNA from fecal samples. Specific primers were designed based on the conserved regions of the BPV VP2 gene in GenBank. The primers for PCR were designed using the Oligo6.0 software based on the conserved regions of VP2 gene. The amplicon sizes were 206 bp. The forward primer sequence is 5′-GCTGGCACTGCCGGGT-3′, and the reverse primer sequence is 5′-CTCCCTCTATTCCTCGGCTCT-3′. The reaction conditions were as follows: 95°C for 5 min followed by 30 cycles of 94°C for 30 s, 45°C for 30 s, and 72°C for 30 s and a final extension at 72°C for 10 min. Products were visualized on 2% agarose gels. The detection results of the signal-enhanced test strips were compared with those obtained using PCR.

## 3. Results

### 3.1. Quality evaluation of purified polyclonal antibodies and monoclonal antibodies

**Figure 2:**
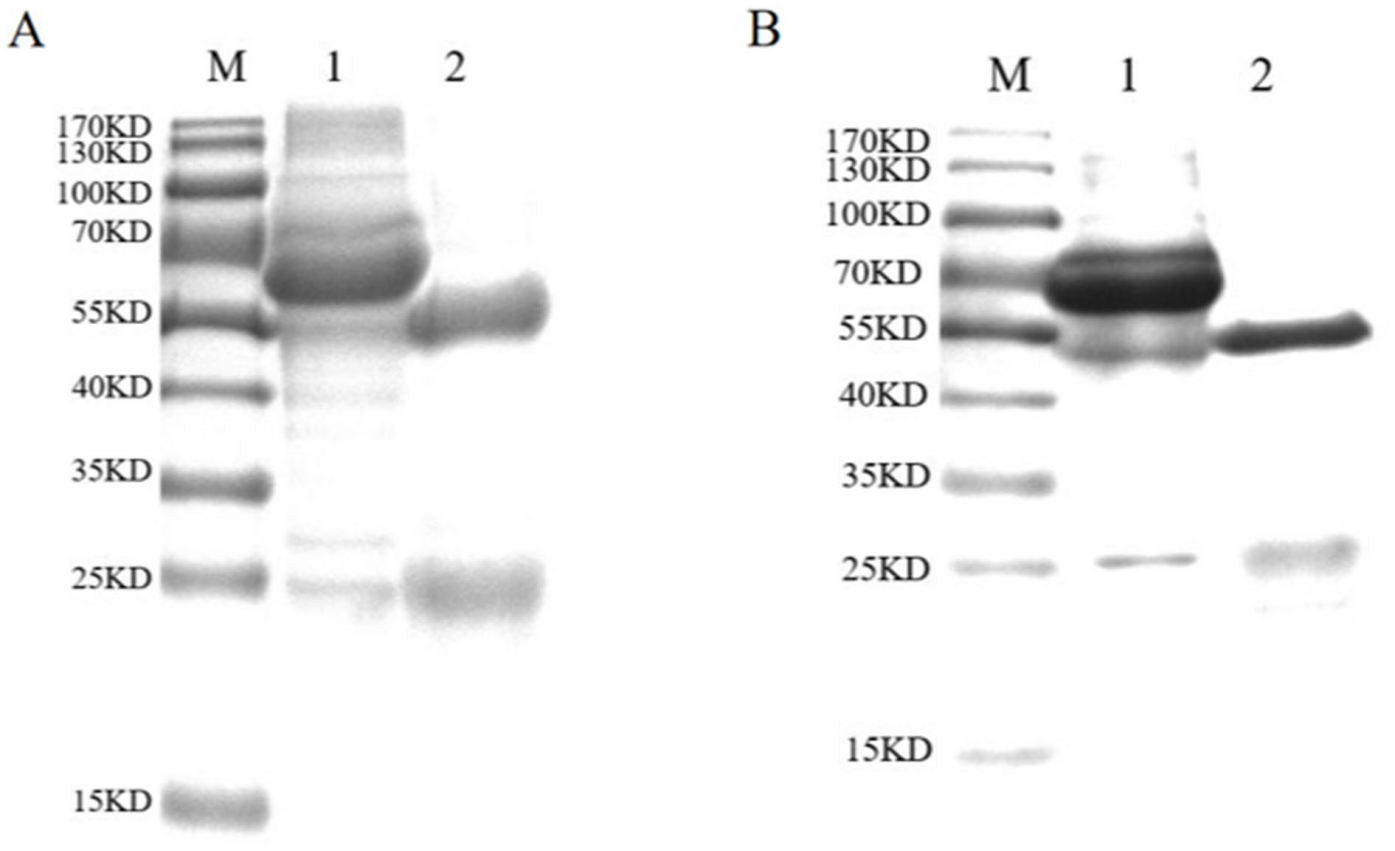
SDS-PAGE analysis of purified antibodies. **(A)** SDS-PAGE analysis of purified pAbs. Lane M: standard protein marker; Lane 1: unpurified pAbs; Lane 2: purified pAbs. The IgG protein was purified effectively. The light chain and heavy chain of IgG were clear and visible, and the sizes of them were 54 and 24 kDa, respectively. **(B)** SDS-PAGE analysis of purified mAbs. Lane M: standard protein marker; Lane 1: unpurified mAbs; Lane 2: purified mAbs; the mAbs were purified effectively. The light chain and heavy chain were clear and visible, and the sizes of them were 55 and 25 kDa, respectively.

**Figure 3:**
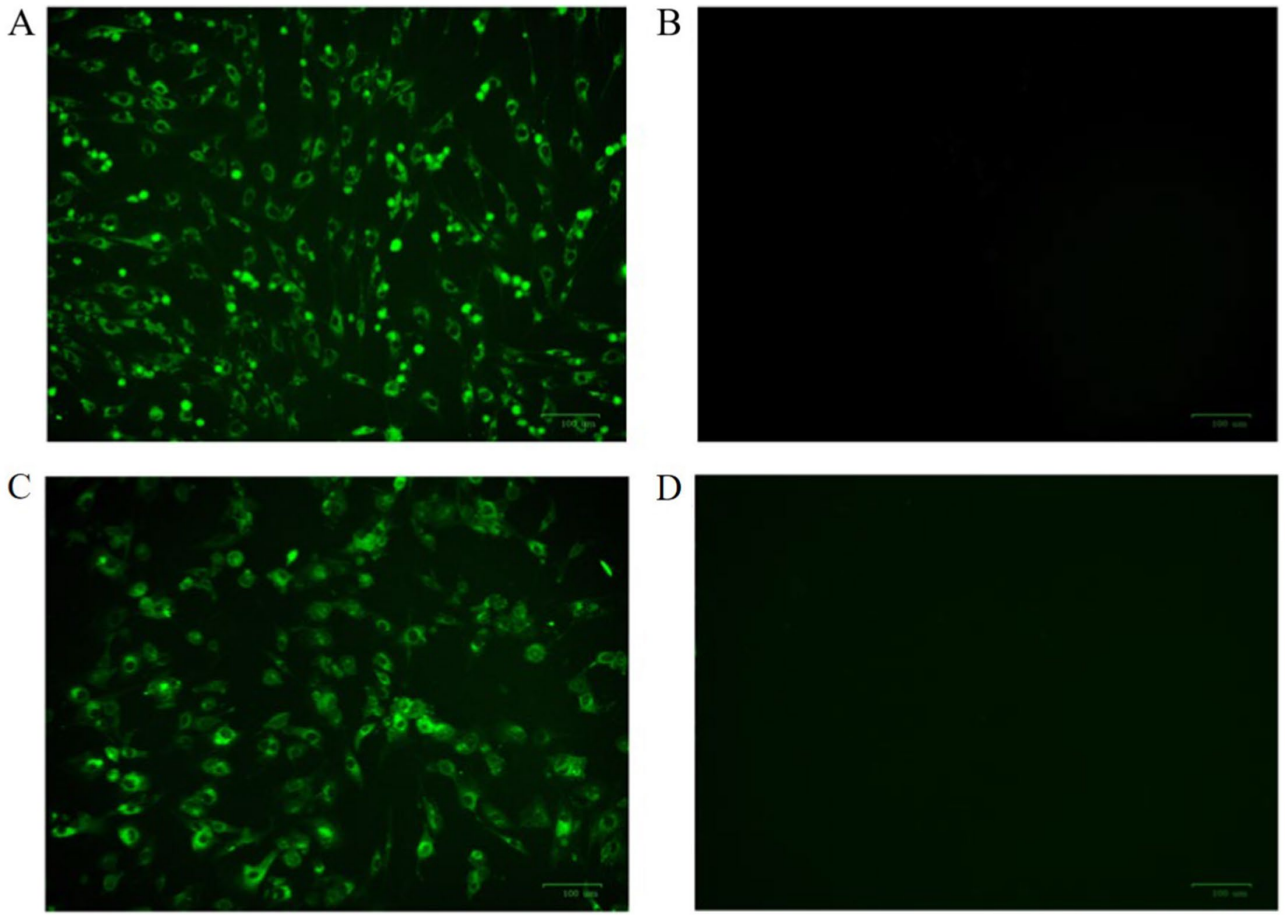
The indirect immunofluorescence identification results of antibodies were observed by fluorescence microscope. **(A)** BPV polyclonal antibody as primary antibody; **(B)** negative serum for primary antibody; **(C)** BPV monoclonal antibody as primary antibody; and **(D)** SP2/0 cell culture supernatant as primary antibody.

Mouse sera were collected 7 days after the last immunization. After purification using standard ammonium sulfate precipitation, aliquots were analyzed *via* SDS-PAGE. The results showed that the IgG protein was effectively purified. The light and heavy chains of IgG were both clear and visible (Figure 2A). The purified BPV pAbs were identified using IFA (Figure 3). The results showed that green fluorescence could be detected in cells treated with BPV pAbs (Figure 3A), while no fluorescence was observed in the negative control (Figure 3B). The protein concentration was measured to be 10.52 mg/mL using a trace protein concentration meter. The pAbs against BPV was coated on the test line as a capture antibody. The pAbs coated on the test line was 10.52 mg/mL.

**Figure 4:**
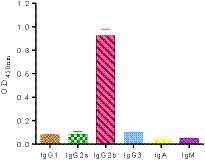
Subclass determination of the monoclonal antibody cells. The results showed that the subtype of monoclonal antibody was IgG2b subtype.

**Figure 5:**
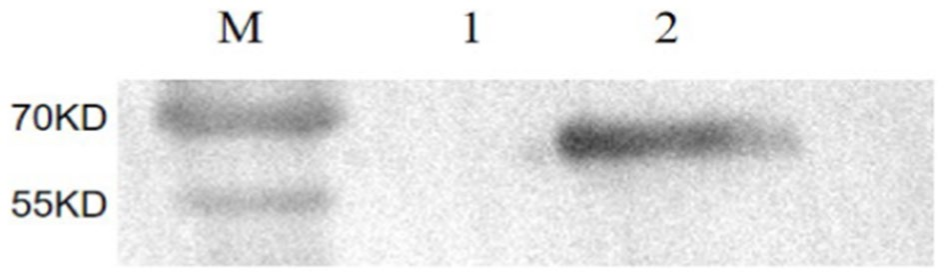
The identification of mAbs by Western blot. M: Protein molecular weight marker; 1: Negative control; 2: Purified BPV.

The purified mAbs were further analyzed using SDS-PAGE and IFA. The results showed that mAbs were effectively purified as both light and heavy chains were clear and visible (Figure 2B). The purified BPV mAbs were further evaluated using IFA. Specific green fluorescence was observed in cells treated with mAbs (Figure 3C), while no fluorescence was observed in the negative control (Figure 3D). The protein concentration was determined to be 3.12 mg/mL as measured using a trace protein concentration meter. The results showed that the subtype of mAbs was IgG2b (Figure 4). Western blot analysis showed that the band size was about 61 kDa, which was consistent with the size of the VP2 protein of BPV (Figure 5).

### 3.2. Characterization of colloidal gold particles

**Figure 6:**
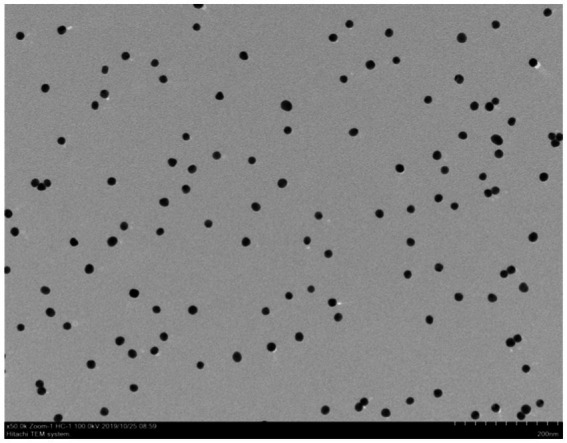
Transmission electron microscopy image of gold nanoparticles. Transmission electron microscope observation showed that particles had varying sizes and shapes with an average diameter of 25–15 nm.

**Figure 7:**
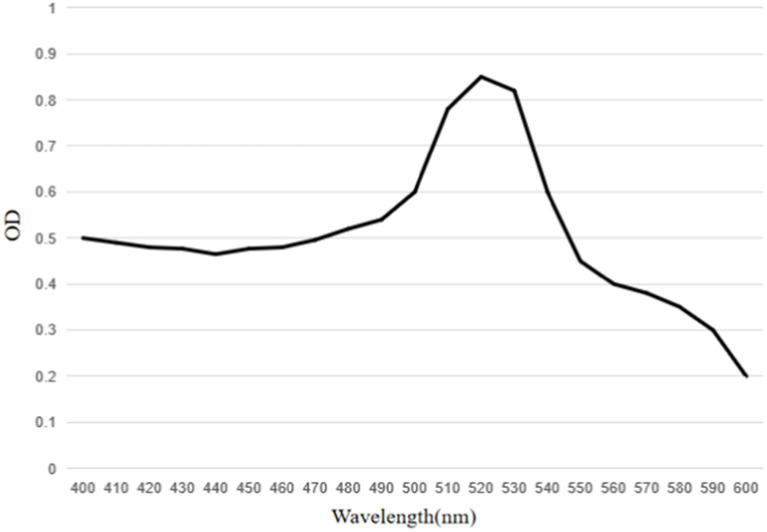
UV–visible spectra of mAbs labeled colloidal gold particles. The peak of the colloidal gold curve was at 520 nm due to surface resonance of the colloidal gold particles. The peak width was narrow and met the standard of colloidal gold to use as probes.

The results showed that the prepared colloidal gold particles were well-dispersed and uniform in size (Figure 6). Colloidal gold particles were spherical, with an average diameter of 18.8–22.5 nm. No aggregation of colloidal gold particles occurred, indicating that colloidal gold particles were stable in the solution. Colloidal gold particles exhibited good stability, and no precipitation occurred within 2 months. The absorption peak was at 520 nm according to the UV/Vis spectra because of the surface resonance of the colloidal gold particles. The peak width was narrow (Figure 7), meeting the standard for using colloidal gold as a probe.

### 3.3. Optimization and characterization of antibody-gold conjugates

**Figure 8:**
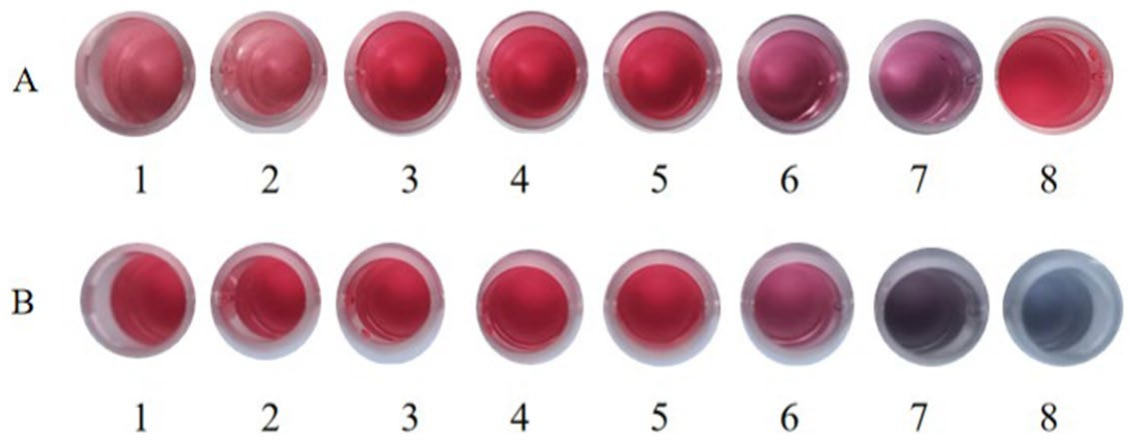
The optional pH of colloidal gold in labeling. **(A)** The colloidal gold solution was added into different tubes (1,000 μL/tube) and the pH values of the colloidal gold solution were adjusted to (1) 7.0, (2) 7.5, (3) 8.0, (4) 8.5, (5) 9.0, (6) 9.5, and (7) 10.0 with 0.2 M K2CO3, respectively. (8) Colloid gold solution without pH adjustment. **(B)** The optimum amount of gold-labeled antibodies. (1) Colloid gold solution. The 1 mL of colloidal gold solution was added quickly into 100 μL of (2) 312.5 μg/mL, (3) 156.25 μg/mL, (4) 78.13 μg/mL, (5) 39.06 μg/mL, (6) 19.53 μg/mL, (7) 9.77 μg/mL, and (8) 0 μg/mL of mAbs, respectively.

The optimum pH value of the colloidal gold solution was the minimum pH value at which the solution remained reddish, which was 8.5 (Figure 8A). At pH 8.5, the optimum concentration of the purified antibody was determined. The result showed that the minimum concentration of purified mAbs to maintain the reddish color of the solution was 39.06 μg/mL (Figure 8B). The optimum concentration of mAbs to be added was 130% of this minimum to stabilize the solution. Hence, the optimum concentration of purified mAbs for conjugation was 50.78 μg/mL.

### 3.4. Characterization of colloidal gold and enzyme-labeled antibody conjugates

**Figure 9:**
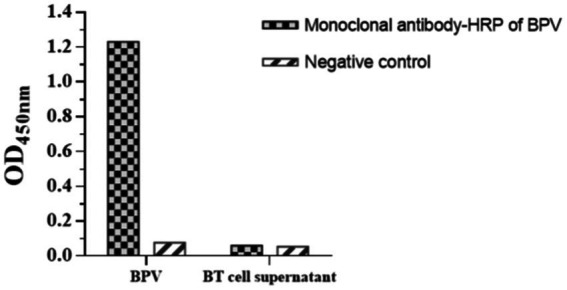
Identification of HRP labeled mAbs against BPV by direct ELISA.

**Figure 10:**
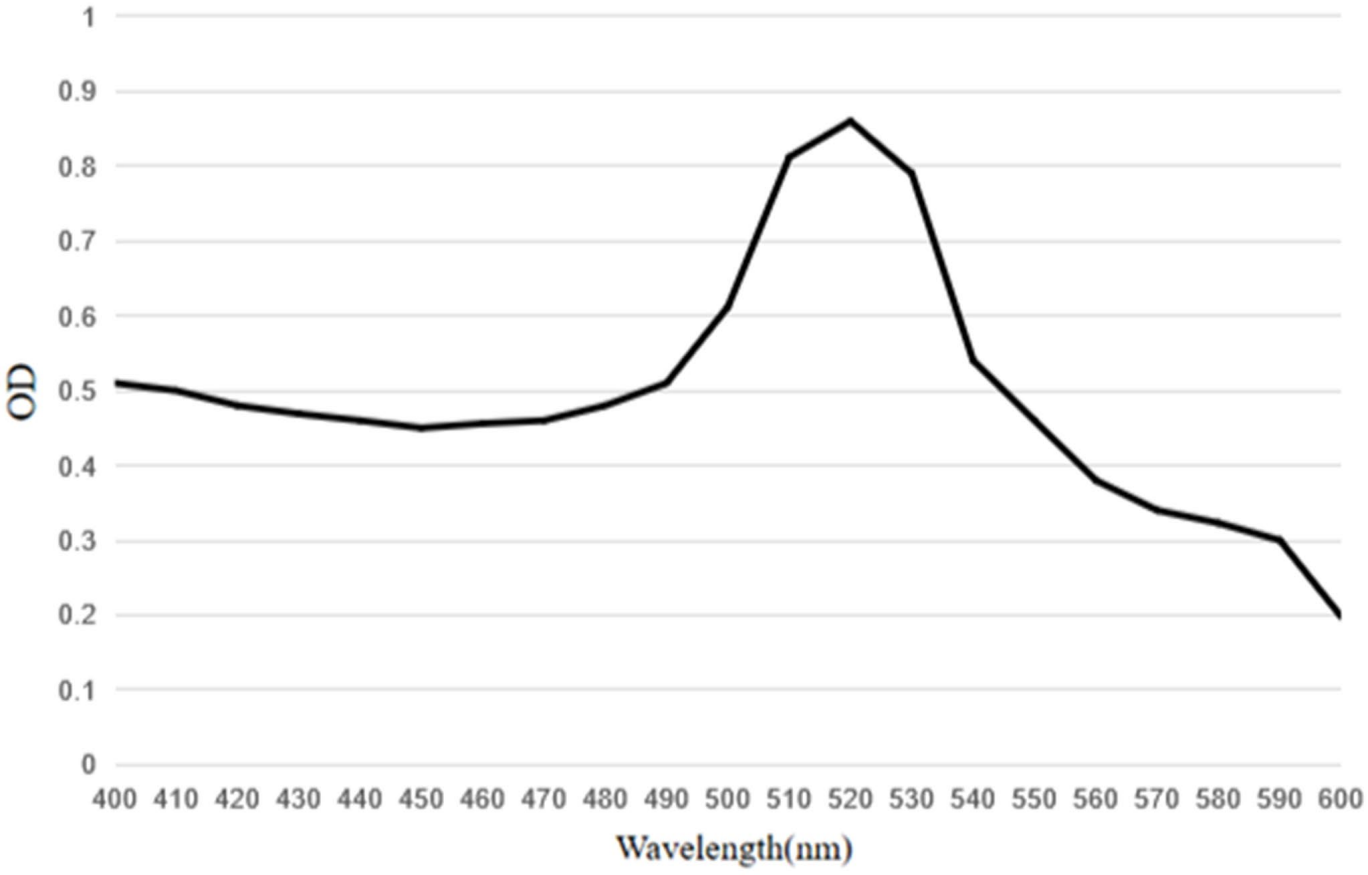
UV–visible spectra of conjugate of mAbs with HRP labeled colloidal gold particles. The peak of the colloidal gold curve was at 520 nm due to surface resonance of the colloidal gold particles. The peak width was narrow and met the standard of colloidal gold to use as probes.

Horseradish peroxidase-labeled mAbs against BPV were detected *via* direct ELISA, and their OD_450_ values were determined (Figure 9). The results showed that HRP-labeled BPV mAbs were successfully prepared. Similarly, the UV/Vis absorption peak was at 520 nm because of the surface resonance of the colloidal gold particles (Figure 10).

### 3.5. Optimization of reaction reagents

**Figure 11:**
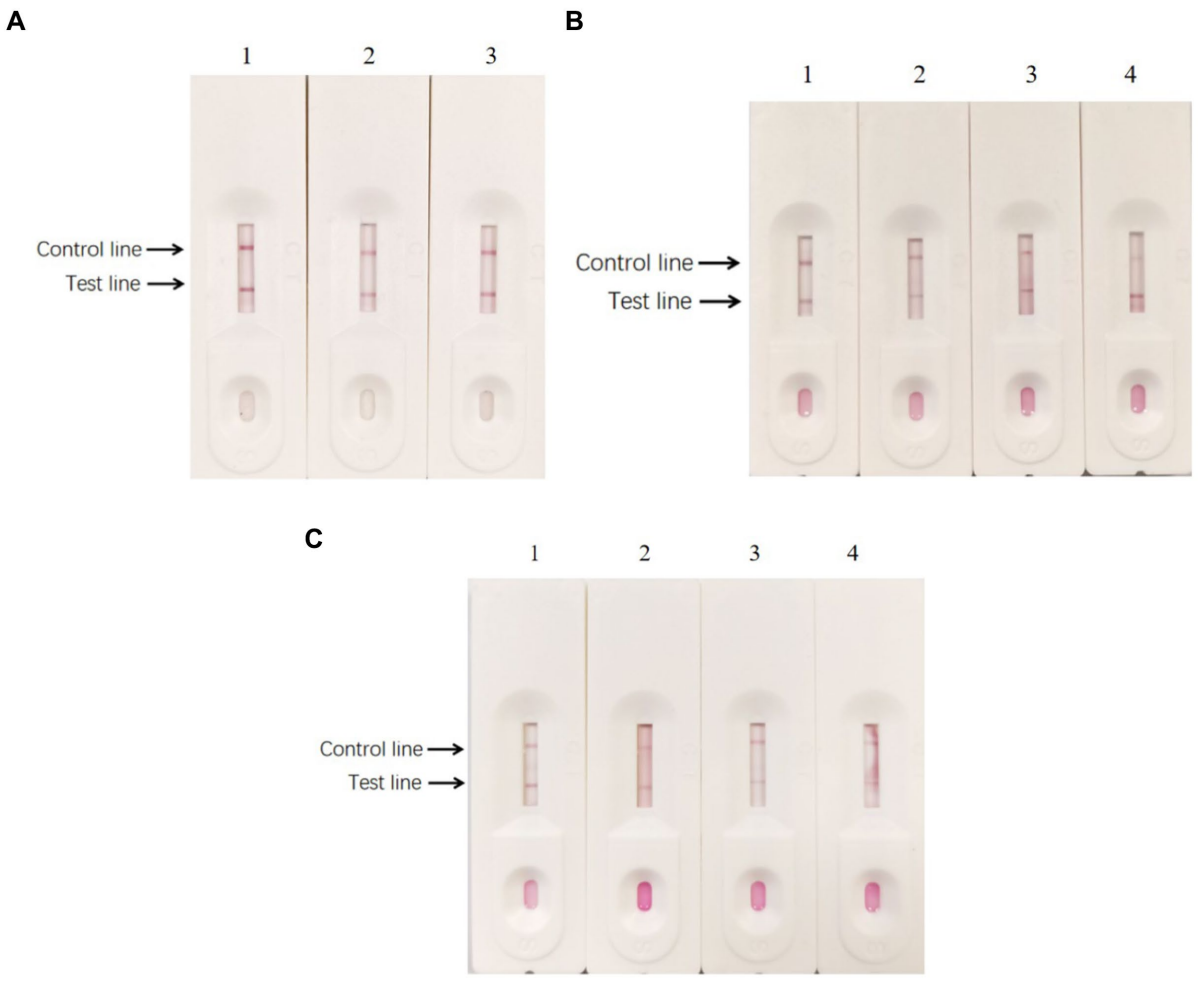
Optimization of reaction reagents. **(A)** Different coating solutions were used to treat the antibodies. (1) 0.01 M PB; (2) PBS, and (3) 20 mM Tris-Cl in treating the antibodies. **(B)** Different blocking solution. (1) 3% BSA; (2) 5% BSA; (3) 3% skim milk, and (d) 5% skim milk. **(C)** The sample pads were soaked in different solutions, respectively. (1) Containing 0.05% Tween-20, 5% sucrose, 0.3% Triton X-100, and 1% BSA; (2) containing 0.05% Tween-20, 5% sucrose, 0.5% Triton X-100, and 0.5% BSA; (3) containing 5% sucrose, 0.3% Triton X-100, and 1% BSA; (4) containing 5% sucrose, 0.5% Triton X-100, and 0.5% BSA.

According to the hybridization method mentioned above, the results showed that 0.01 M PB was the most effective coating solution in treating antibodies. The bands of the control line and the test line were more clearly visible after treating the antibodies with 0.01 M PB (Figure 11A). Furthermore, 3% BSA, 5% BSA, 3% skim milk, and 5% skim milk solution were used as blocking solutions (Figure 11B). The background was lighter when 3% BSA and 5% BSA solutions were used for blocking compared to the other solutions. Furthermore, the blocking time was shorter when using 3% BSA compared to 5% BSA, which was more suitable for rapid testing. The sample pads were soaked in different treatment solutions. The results showed that the test line was most clearly visible when the sample pad was soaked in solution A (0.05% Tween-20, 5% sucrose, 0.3% Triton X-100, and 1% BSA; Figure 11C). Based on these results, solution A was used to soak the sample pads.

### 3.6. Sensitivity and specificity evaluation

**Figure 12:**
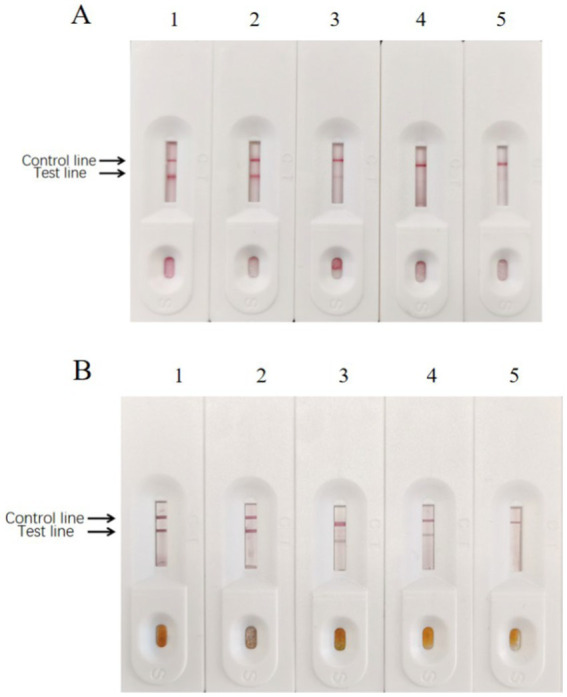
Sensitivity of enzyme-labeled signal-enhanced test strips. **(A)** Sensitivity of traditional GICG test strip. **(B)** Sensitivity of signal enhanced GICG test strip. BPV of 10^5^ TCID_50_/0.1 ml was diluted in (1) 1, (2) 10, (3) 100, (4) 1000 and (5) 10000 fold, respectively.

After preparing the conventional test strips, the sensitivity test results showed that the minimum amount of BPV they detected was approximately 10^3^ TCID_50_ (Figure 12A). In contrast, the sensitivity test results of the signal-enhanced test strips showed that the minimum amount of BPV they detected was approximately 10^2^ TCID_50_ (Figure 12B). These results indicate that the sensitivity of the signal-enhanced test strip was 10 times higher than that of an conventional test strip.

**Figure 13:**
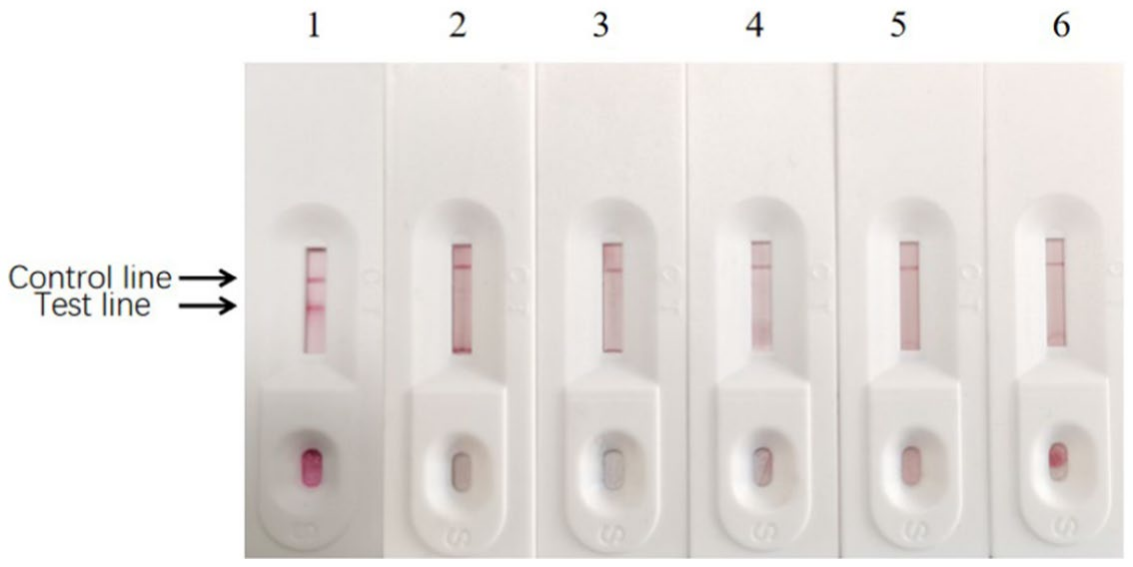
Results of specificity test of enzyme-labeled signal-enhanced test strips. (1) BPV; (2) BRV; (3) BVDV; (4) BRSV; (5) BT cell culture; (6) PB buffer solution.

The specificity test results showed no cross-reaction when detecting BRV, BVDV, and BRSV using signal-enhanced test strips, showing good specificity (Figure 13). These results suggest that the signal-enhanced test strips showed good reactivity and specificity in detecting BPV.

### 3.7. Clinical sample testing

**Figure 14:**
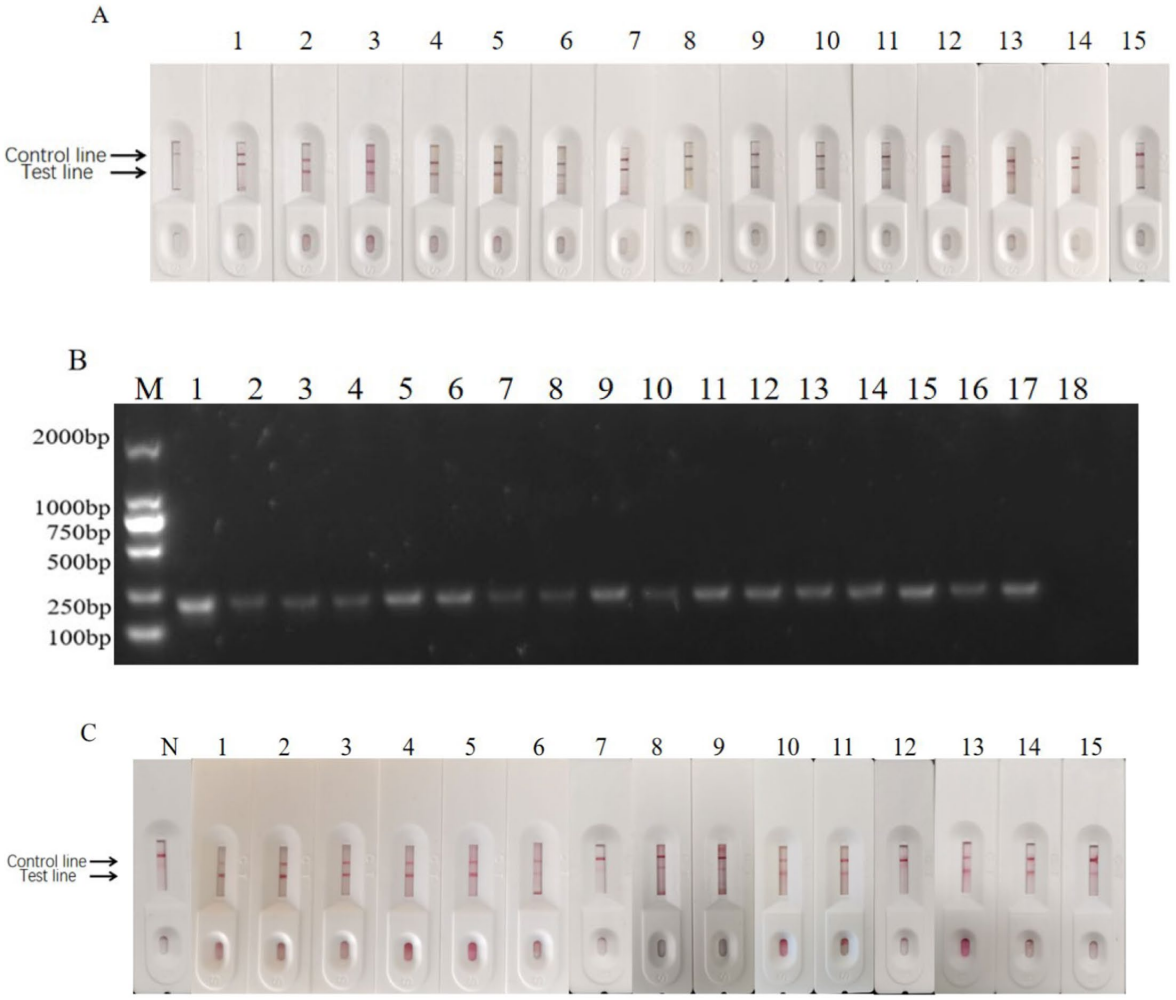
Clinical sample test results. **(A)** Positive sample test results of enzyme-labeled signal-enhanced test strips. N: Negative control; 1: Heilongjiang No.10; 2: Heilongjiang No.41; 3: Heilongjiang No.36; 4: Heilongjiang No.52; 5: Heilongjiang No.6; 6: Heilongjiang No.5; 7: Heilongjiang No.66; 8: Heilongjiang No.50; 9: Neimenggu No. 36; 10: Jilin No.8; 11: Jilin No.6; 12: Jilin No.22; 13: Heilongjiang No.235; 14: Heilongjiang No.231; and 15: Positive control. **(B)** The detection result of positive samples by PCR. M: DNA Marker; 1: Positive control; 2: Heilongjiang No.41; 3: Heilongjiang No.61; 4: Heilongjiang No.36; 5: Heilongjiang No.52; 6: Heilongjiang No.6; 7: Heilongjiang No.5; 8: Heilongjiang No.3; 9: Heilongjiang No. 66; 10: Heilongjiang No.50; 11: Neimenggu No.36. 12: Jilin No.8. 13: Jilin No.6. 14: Heilongjiang No.10; 15: Jilin No.22; 16: Heilongjiang No.235; 17: Heilongjiang No.231; and 18: Negative control. **(C)** Positive sample test results of enzyme-labeled signal-enhanced test strips. N: Negative control; 1: Heilongjiang No.10; 2: Heilongjiang No.41; 3: Heilongjiang No.36; 4: Heilongjiang No.52; 5: Heilongjiang No.6; 6: Heilongjiang No.5; 7: Heilongjiang No.66; 8: Heilongjiang No.50; 9: Neimenggu No. 36; 10: Jilin No.8; 11: Jilin No.6; 12: Jilin No.22; 13: Heilongjiang No.235; 14: Heilongjiang No.231; and 15: Positive control.

Two hundred and eighty fecal samples were collected from different farms (Jilin, Liaoning, and Heilongjiang provinces). Each sample was detected using the signal-enhanced GICG strip (Figure 14A) and PCR (Figure 14B). Fourteen positive samples were detected using the signal-enhanced GICG strip. Twelve positive samples were detected using the conventional GICG strip, while 16 positive samples were detected by PCR. The test results for the signal-enhanced GICG strip and PCR were then compared (Table 1), showing that the concordance rate between the signal-enhanced GICG strip and PCR was 99.29%.The test results for the conventional GICG strip and PCR were then compared (Table 1), showing that the concordance rate between the conventional GICG strip and PCR was 98.57%. These results indicate that the sensitivity of the signal-enhanced test strip was higher than that of an conventional test strip.

**Table 1:** Test results of clinical samples by signal-enhanced GICG strips and PCR.

Test method	Number of samples	Detection result (negative/positive)	Compliance rate of signal enhanced GICG strip	Positive sample compliance rate of signal enhanced GICG strip	Negative samples conformity rate of signal enhanced GICG strip
The signal enhanced GICG strip	280	266/14			
PCR	280	264/16	99.29%	87.5%	100%
The conventional GICG strip	280	268/12	99.29%	85.71%	100%

## 4. Discussion

Bovine parvovirus infections can cause abortions in pregnant cows and affect herd reproduction rates (Barnes et al., 1982). Calves infected with BPV show respiratory and digestive symptoms such as dyspnea, cough, and diarrhea. After pathological examination of their respiratory and digestive tracts, lesions of different degrees were observed. BPV infection can be transmitted vertically and horizontally. Pregnant cows can directly infect fetal cattle during pregnancy (Liggitt et al., 1982). The seroprevalence of BPV in cattle herds is high owing to multiple transmission routes. Moreover, the initial clinical signs are not obvious as recessive infections (Storz et al., 1978), making early diagnosis and prevention difficult.

Some etiological and serological techniques have been developed for detecting BPV infections, such as virus isolation and identification, electron microscopy, PCR, fluorescence quantitative PCR (FQ-PCR), serum neutralization tests, IFA, and ELISA. Most of these methods need to be performed in the laboratory and require technicians or special instruments (Mengeling et al., 1986; Joon et al., 2019; Wang et al., 2019b). Therefore, these methods are unsuitable for field testing.

As a rapid and simple detection method, GICG technology has been increasingly used to detect various infectious pathogens (Song et al., 2016; Yu et al., 2019). BPV-infected cattle can shed viral particles in their feces; therefore, it is possible to detect antigens in fecal samples. In the present study, we developed a signal-enhanced GICG containing mAbs and pAbs against BPV to detect fecal antigens. Colloidal gold particles can initially adsorb protein antibodies (Pollitt et al., 2015). Many studies have shown that colloidal gold particles approximately 20 nm in diameter are suitable for mAb labeling. Hence, colloidal gold particles of approximately 20 nm in diameter were prepared in this study, which were dispersed and uniform in size. The dispersed colloidal gold particles flowed easily through the membrane. Moreover, the prepared colloidal gold particles had good stability, and no precipitation occurred within 2 months. The reaction conditions of the immunochromatographic test strip are important parameters affecting its quality (Ge et al., 2018; Li et al., 2020). Here, we optimized different reaction conditions, including the amount of mAbs, pH of the colloidal gold solution, coating solution, blocking solution, sample pad treatment solution, antibody concentration in the control line, and antibody concentration in the detection line. The method we developed is based on the double-antibody sandwich ELISA principle. Polyclonal antibodies, as capture antibodies, can recognize a variety of antigen epitopes. The mAbs against BPV are conjugated to HRP as the detection antibody, which can amplify the detection signal.

Most GICG tools for pathogen detection are based on the generation of color signals from colloidal gold tracers that are visible to the naked eye. These methods often exhibit low sensitivity. New materials have been introduced to improve the sensitivity of these test strips. Parolo et al. (2013) developed a GICG with a detection antibody modified with HRP, which amplified the detection signal. Cho et al. (2015) developed an enhanced enzyme-labeled GICG system. The detection limit of this system for *Escherichia coli* O157:H7 was 100 CFU/mL, which increased approximately 1,000-fold due to signal amplification (Cho et al., 2015). In this study, enzyme-labeled mAbs were used instead of mAbs on the test line to improve the sensitivity of the strip test. We used mAbs and enzyme-labeled mAbs to prepare the signal-enhanced colloidal gold test strips. The limit of detection for BPV was 10^2^ TCID_50_/0.1 mL using the developed test strip. These results indicate that the sensitivity of the signal-enhanced test strip was 10 times higher than that of an ordinary test strip. TMB was used as an insoluble blue-violet chromogen, which was deposited in the control and test lines. The TMB introduced in this test did not require the use of a professional luminescence analyzer, and the improvement in sensitivity could be observed with the naked eye.

The validation assay revealed that the prepared test strip showed high specificity and no cross-reactivity with other clinical pathogens. To detect clinical samples, we compared the results of the test strip and PCR. The two methods showed good correspondence. Moreover, the test strips exhibited good specificity. Although PCR has a higher sensitivity and lower detection limit, the test strip is both simple and rapid. Unlike conventional detection methods, colloidal gold-based immunochromatographic strips can be easily used without special equipment and only require simple visual judgment. Therefore, the colloidal gold immunochromatographic method established in this study not only improves the sensitivity of test strips but also does not require special detection instruments for diagnosing BPV infections. In the future, the GICG strip developed in this study may be more suitable for practical production, particularly for large-scale field analyses.

## Data availability statement

The original contributions presented in the study are included in the article/supplementary material, further inquiries can be directed to the corresponding author.

## Ethics statement

The animal study was reviewed and approved by Ethical Committee for Animal Sciences of Heilongjiang Province Ethics Committee for Laboratory Animals of Northeast Agricultural University.

## Author contributions

XY, SZ, and CW performed the experiments. RW and LZ analyzed the experimental data. ST, WC, and JL collected the clinical samples. YJ collected the clinical samples and contributed to manuscript revision. XQ conceived and designed the study. All authors contributed to the article and approved the submitted version.

## Funding

This research was funded by Key Research and Development Program of Heilongjiang Province (Grant no: GA21B004), the Natural Science Foundation of Heilongjiang Province (Grant no: LH2020C022), and the National Natural Science Foundation of China (NSFC; No. 32072876).

## Conflict of interest

The authors declare that the research was conducted in the absence of any commercial or financial relationships that could be construed as a potential conflict of interest.

## Publisher’s note

All claims expressed in this article are solely those of the authors and do not necessarily represent those of their affiliated organizations, or those of the publisher, the editors and the reviewers. Any product that may be evaluated in this article, or claim that may be made by its manufacturer, is not guaranteed or endorsed by the publisher.

## References

[ref1] Barnes M. A. Wright R. E. Bodine A. B. Alberty C. F. (1982). Frequency of bluetongue and bovine parvovirus infection in cattle in South Carolina dairy herds. Am. J. Vet. Res. 6, 1078–1080. 6285770

[ref2] Cho I. H. Bhunia A. Irudayaraj J. (2015). Rapidpathogendetectionbylateral-flowimmunochromatographicassaywith gold nanoparticle-assisted enzyme signal amplification. Int. J. Food Microbiol. 206, 60–66. doi: 10.1016/j.ijfoodmicro.2015.04.032, PMID: 25955290

[ref3] Ge W. L. Suryoprabowo S. Wu X. L. Zheng Q. K. Kuang H. (2018). Rapid immunochromatographic test strip detection of mabuterol and its cross-reactivity with mapenterol. Food Agric. Immunol. 29, 1028–1040. doi: 10.1080/09540105.2018.1499709

[ref4] Gong Z. D. Shen X. Y. Liang H. Q. Geng J. J. Wei S. C. (2020). Taq Manprobeq RT-PCRdetectsbovineparvovirus and appliesclinically. Turk. J. Vet. Anim. Sci. 44, 364–369. doi: 10.3906/vet-1907-80

[ref5] Guo J. C. Chen S. Q. Guo J. H. Ma X. (2021). Nanomaterial Labelsin LateralFlowImmunoassaysforPoint-of-care-testing. J. Mater. Sci. Technol. 60, 90–104. doi: 10.1016/j.jmst.2020.06.003

[ref6] Guo W. S. Zhang Y. Hu X. Y. Zhang T. Liang M. Yang X. L.. (2019). Region growing AlgorithmCombinedWithFastPeakDetectionforSegmentingColloidalGoldImmunochromatographicStripImages. IEEE Access 7, 169715–169723. doi: 10.1109/ACCESS.2019.2955510

[ref7] Joon D. Nimesh M. Gupta S. Kumar C. Varma-Basil M. Saluja D. (2019). Development and evaluation of rapid and specific sdaA LAMP-LFD assay with Xpert MTB/RIF assay for diagnosis of tuberculosis. J. Microbiol. Methods 159, 161–166. doi: 10.1016/j.mimet.2019.03.002, PMID: 30858005

[ref8] Kailasan S. Halder S. Gurda B. Bladek H. Chipman P. R. McKenna R.. (2015). Structure of an enteric pathogen, bovine parvovirus. J. Virol. 89, 2603–2614. doi: 10.1128/JVI.03157-14, PMID: 25520501 PMC4325758

[ref9] Li G. Q. Rong Z. Wang S. Q. Zhao H. Y. Piao D. R. Yang X. W.. (2020). Rapid detection of brucellosis using a quantum dot-based immunochromatographic test strip. PLos Neglect Trop. 14:e0008557. doi: 10.1371/journal.pntd.0008557, PMID: 32976512 PMC7540878

[ref10] Liggitt H. D. DeMartini J. C. Pearson L. D. (1982). Immunologic responses of the bovine fetus to parvovirus infection. Am. J. Vet. Res. 8, 1355–1359. 6980610

[ref11] Lin L. Wu X. L. Cui G. Song S. S. Kuang H. Xu C. L. (2020). Colloidal gold ImmunochromatographicStripAssayfortheDetectionofAzaperoneinPork and pork liver. ACS Omega 5, 1346–1351. doi: 10.1021/acsomega.9b01841, PMID: 32010804 PMC6990434

[ref12] Mengeling W. L. Paul P. S. Bunn T. O. Ridpath J. F. (1986). Antigenic relationships among autonomous parvoviruses. J. Gen. Virol. 67, 2839–2844. doi: 10.1099/0022-1317-67-12-2839, PMID: 2432167

[ref13] Pan Y. B. Li X. R. Yang G. Fan J. L. Tang Y. T. Zhao J.. (2020). Serologicalimmunochromatographicapproachindiagnosiswith SARS-CoV-2infectedCOVID-19patients. J. Inf. Secur. 81, E28–E32. doi: 10.1016/j.jinf.2020.03.051, PMID: 32283141 PMC7195339

[ref14] Parolo C. de la Escosura-Muniz A. Merkoci A. (2013). Enhancedlateralflowimmunoassayusinggoldnanoparticles loaded with enzymes. Biosens. Bioelectron. 40, 412–416. doi: 10.1016/j.bios.2012.06.049, PMID: 22795532

[ref15] Pollitt M. J. Buckton G. Piper R. Brocchini S. (2015). Measuring antibody coatings on gold nanoparticles by optical spectroscopy. RSC Adv. 5, 24521–24527. doi: 10.1039/c4ra15661g

[ref16] Qiu J. M. Soderlund-Venermo M. Young N. S. (2017). Human Parvoviruses. Clin. Microbiol. Rev. 30, 43–113. doi: 10.1128/CMR.00040-16, PMID: 27806994 PMC5217800

[ref17] Shukla S. Leem H. Kim M. (2011). Development of a liposome-based immunochromatographic strip assay for the detection of Salmonella. Anal. Bioanal. Chem. 401, 2581–2590. doi: 10.1007/s00216-011-5327-2, PMID: 21863217

[ref18] Song C. M. Liu C. Wu S. Y. Li H. L. Guo H. Q. Yang B.. (2016). Development of a lateral flow colloidal gold immunoassay strip for the simultaneous detection of Shigella boydii and Escherichia coli O157:H7 in bread, milk and jelly samples. Food Control 59, 345–351. doi: 10.1016/j.foodcont.2015.06.012

[ref19] Storz J. Young S. Carroll E. J. Bates R. C. Bowen R. A. Keney D. A. (1978). Parvovirus infection of the bovine fetus:distribution of infection, antibody response，and age-related susceptibility. Am. J. Vet. Res. 7, 1099–1102. 677527

[ref20] Vilela D. Gonzalez M. C. Escarpa A. (2012). Sensingcolorimetricapproachesbasedongold and silvernanoparticlesaggregation: Chemicalcreativitybehindtheassay. Anal. Chim. Acta 751, 24–43. doi: 10.1016/j.aca.2012.08.043, PMID: 23084049

[ref21] Wang Y. Wang L. F. Zhang J. W. Wang G. X. Chen W. B. Chen L.. (2014). Preparation of colloidal gold immunochromatographic strip for detection of paragonimiasis skrjabini. PLoS One 9:e092034. doi: 10.1371/journal.pone.0092034, PMID: 24643068 PMC3958401

[ref22] Wang M. M. Yan Y. Wang R. C. Wang L. Zhou H. Li Y. J.. (2019b). Simultaneous detection of bovine rotavirus, bovine parvovirus, and bovine viral diarrhea virus using a gold nanoparticle-assisted PCR assay with a dual-priming oligonucleotide system. Front. Microbiol. 10:2884. doi: 10.3389/fmicb.2019.02884, PMID: 31921061 PMC6920155

[ref23] Wang M. M. Yan Y. Wang R. C. Wang L. Zhou H. Li Y. J.. (2019a). Simultaneous Detectionof BovineRotavirus, bovine parvovirus, and bovine viral DiarrheaVirusUsingaGoldNanoparticle-assisted PCRAssayWithaDual-priming oligonucleotide system. Front. Microbiol. 10:2884. doi: 10.3389/fmicb.2019.02884, PMID: 31921061 PMC6920155

[ref24] Wu W. D. Li M. Chen M. Li L. P. Wang R. Chen H. L.. (2017). Developmentofacolloidalgoldimmunochromatographicstripforrapiddetectionof Streptococcusagalactiaeintilapia. Biosens. Bioelectron. 91, 66–69. doi: 10.1016/j.bios.2016.11.038 27992801

[ref25] Yu M. M. Bao Y. L. Wang M. P. Zhu H. B. Wang X. Y. Xing L. X.. (2019). Development and application of a colloidal gold test strip for detection of avian leukosis virus. Appl. Microbiol. Biotechnol. 103, 427–435. doi: 10.1007/s00253-018-9461-z, PMID: 30349931

[ref26] Zhang M. D. Hill J. E. Godson D. L. Ngeleka M. Fernando C. Huang Y. Y. (2020). Thepulmonaryvirome, bacteriological and histopathologicalfindingsinbovinerespiratorydiseasefromwestern Canada. Transbound. Emerg. Dis. 67, 924–934. doi: 10.1111/tbed.13419, PMID: 31715071 PMC7168541

